# A Novel SimpleDrop Chip for 3D Spheroid Formation and Anti-Cancer Drug Assay

**DOI:** 10.3390/mi12060681

**Published:** 2021-06-10

**Authors:** Xiaoli Liu, Huichao Lin, Jiaao Song, Taiyi Zhang, Xiaoying Wang, Xiaowen Huang, Chengyun Zheng

**Affiliations:** 1 Department of Hematology, The Second Hospital, Cheeloo College of Medicine, Shandong University, Jinan 250033, China; liuxiaoli5116@126.com; 2 State Key Laboratory of Biobased Material and Green Papermaking, Department of Bioengineering, Qilu University of Technology (Shandong Academy of Sciences), Jinan 250300, China; linhuichao2020@gmail.com (H.L.); songjiaao0419@126.com (J.S.); zty2020bio@126.com (T.Z.); 3 Department of Pathology, The Second Hospital, Cheeloo College of Medicine, Shandong University, Jinan 250033, China; wang_xy1896@163.com

**Keywords:** cell culture, 3D cell spheres, cancer, drug assay, micro-hole culture chip

## Abstract

Cell culture is important for the rapid screening of anti-cancer drug candidates, attracting intense interest. Traditional 2D cell culture has been widely utilized in cancer biological research. However, 3D cellular spheroids are able to recapitulate the in vivo microenvironment of tissues or tumors. Thus far, several 3D cell culture methods have been developed, for instance, the hanging drop method, spinner flasks and micropatterned plates. Nevertheless, these methods have been reported to have some disadvantages, for example, medium replacement is inconvenient or causes cellular damage. Here, we report on an easy-to-operate and useful micro-hole culture chip (SimpleDrop) for 3D cellular spheroid formation and culture and drug analysis, which has advantages over the traditional method in terms of its ease of operation, lack of shear force and environmentally friendliness. On this chip, we observed the formation of a 3D spheroid clearly. Three drugs (paclitaxel, cisplatin and methotrexate) were tested by both cell viability assay and drug-induced apoptotic assay. The results show that the three drugs present a similar conclusion: cell viability decreased over time and concentration. Moreover, the apoptotic experiment showed a similar trend to the live/dead cell assay, in that the fraction of the apoptotic and necrotic cells correlated with the concentration and time. All these results prove that our SimpleDrop method is a useful and easy method for the formation of 3D cellular spheroids, which shows its potential for both cell–cell interaction research, tissue engineering and anticancer drug screening.

## 1. Introduction

Cancer is one of the most dangerous diseases, with high mortality and morbidity [1,2]. Although researchers invest a large amount of money in cancer research and drug discovery/development, the approval rate for novel drugs is disappointing (5%) and most metastatic cancers are relapse-prone [3,4]. Some 95% of new drugs for cancer therapy fail in clinical development because they present a low success rate and show poor therapeutic effects and/or unacceptable toxicity. One of the main reasons for this is that preclinical models fail to fully simulate the complexity and heterogeneity of human cancers [5,6,7]. In order to verify the effectiveness of drugs, the traditional two-dimensional (2D) cellular monolayer has been successfully and widely utilized, which has been proven to be one of the most convenient and stable systems for cancer biological research, especially for the rapid screening of anti-cancer drug candidates [8,9]. However, with the continuous development of research in anti-cancer fields, the shortcomings of 2D cell culture technology have been gradually exposed. More specifically, 2D cellular growth as a monolayer on a flat solid surface lacks the cell–cell and cell–matrix interactions which exist in primary tumors [10,11,12]. In addition, 2D cultured cells are stretched and undergo cytoskeletal rearrangement, leading to the abnormal expression of genes and proteins caused by artificial polarity [13,14,15,16].

Unlike a traditional 2D cellular monolayer, 3D cell spheres simulate the real heterogeneity and microenvironment of tissue and provide cell–cell and cell–extracellular-matrix interaction information, which supplies a new method for cell–cell signal transduction, drug development and the validation of molecular targets [17,18,19,20]. Thus far, several 3D cell culture methods have been developed. For instance, the hanging drop method [21], spinner flasks [22] and micropatterned plates [23].

However, these methods have been reported to have some disadvantages [24,25]. In terms of the hanging drop method, (1) the cell growth space is narrow, (2) medium replacement is inconvenient (for example, during the medium replacement process, if the Petri dish is upright, cell spheres are easy to contact with the culture plate and further grow adherently on the Petri dish; if the Petri dish is downwards, randomly distributed cell spheres are easily aspirated mistakenly and come into contact with other culture droplets), and (3) drug administration or factor addition to the culture medium is difficult for similar reasons as with medium replacement [26]. In terms of the spinner flask method, a high stirring rate may result in high shear force and damage to 3D cell spheres. However, an extremely slow stirring speed will cause the spherical cells to sink to the bottom of the container, thereby inhibiting the formation of spherical cells in the container [27,28]. Besides this, the micropatterning method requires specialized chemical techniques and photosensitive materials for the preparation of micropattern structures, and has the drawbacks of biologically incompatible and complex processes [29]. Various works were attempted in order to overcome the above shortcomings. Tung et al. reported a 384-well format hanging-drop culture plate, which proved that 3D culture was more suitable for tirapazamine (TPZ) [30]. Patra et al. reported a two-layered microfluidic device that controlled the size of tumor spheroids by adjusting the geometry of the cell culture chamber [31]. Chen et al. developed a microfluidic sphere formation platform using a novel polyHEMA (non-adherent polymer) fabrication process to characterize the different responses in 2D and 3D cell culture to photodynamic therapy (PDT) [32]. Ji An et al. proposed an adjustable spheroid culture platform arrayed with interfacial elastomeric wells. The tumor spheroids formed in the interfacial holes would serve to observe drug responses and monitor reactive oxygen species (ROS) [33]. Dadgar et al. reported a multichamber microfluidic device with integrated microvalves to continuously seed several chambers and then test several drug concentrations in parallel [34]. Liao et al. designed a 3D-printed, reusable, signet-like resin mold which can mold microstructures for the spheroid culture of tumor cells on the surface of agarose strome in a 96-well plate [35].

**Figure 1:**
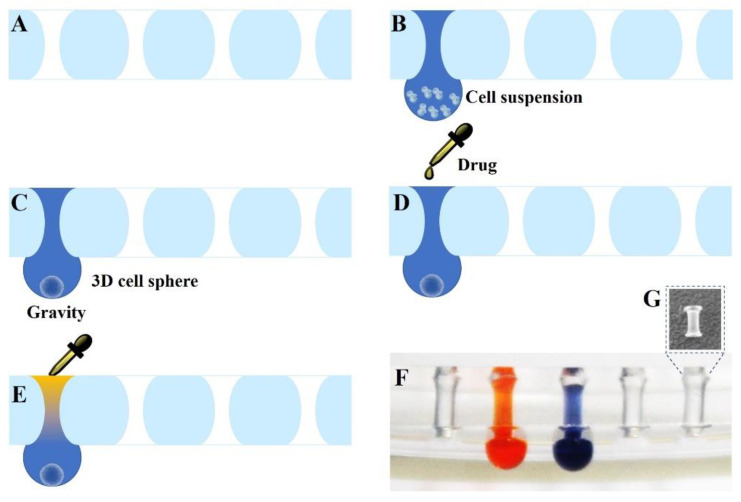
Diagram of the SimpleDrop chip. (**A**) The micro-hole array on a PDMS layer (3 mm thickness) punched by a flat needle. (**B**) Through the micro-holes, cell suspension forms a droplet. (**C**) Cells in the droplet spontaneously aggregate into spheres. (**D**,**E**) Drugs with different concentrations are added from the micro-holes for drug analysis. (**F**) Photographs of the SimpleDrop chip; the holes are waist type with wide ends and a narrow middle. (**G**) PDMS plug.

Here, we report on a micro-hole culture chip (SimpleDrop) for 3D cell spheroid formation, culture and drug analysis. As shown in Figure 1, we punched the micro-hole array on a PDMS layer (3 mm thickness) with a flat needle. Due to the elasticity and deformation of PDMS, the holes were waist type with wide ends and a narrow middle (Figure 1F,G). Through the micro-holes, a cell suspension, culture medium or drug solution could be introduced easily and form a drop under the PDMS layer simultaneously (Figure 1). To avoid liquid evaporation, the culture chip was put into a humid incubator. Subsequently, the cells in the droplet spontaneously aggregated into spheres. Drugs with a different concentration could be added from the micro-holes for drug analysis, and the cytotoxicity could be observed using the fluorescent dying method. On this micro-hole culture chip, we could clearly observe the viability of the cells through a fluorescent signal, which is especially useful for drug analysis. Moreover, PDMS has good biocompatibility and good gas permeability, which is beneficial for gas exchange during cell growth [36,37,38]. Cell culture on the SimpleDrop chip has better controllability and flexibility, which can allow for the precise positioning of target cells through precise control of the chip. The cells can also be stimulated with a precise dose of stimulators, making experiments more accurate and reproducible. We believe this study will contribute to building in vitro models of 3D multicellular tumor spheroids, which could provide promising tools for in vitro drug screening and basic cancer cell research.

## 2. Materials and Methods

### 2.1. Cell Culture

Experiments were performed based on the synovial sarcoma cell line HS-SY-II. The cells were established by Changliang Peng [39] and cultured in Dulbecco’s Modified Eagle’s Medium (DMEM) supplemented with 10% fetal bovine serum (FBS) plus 0.1 mg/mL streptomycin sulfate and 1% penicillin–streptomycin (Life Technologies, Carlsbad, CA, USA). Human umbilical cord derived mesenchymal stem cells (UC-MSCs) were isolated and cultured. Cells were passaged using a trypsin-EDTA solution when they reached 70–80% confluency.

### 2.2. Fabrication of the SimpleDrop Chip

The fabrication of the SimpleDrop chip was uncomplicated. A flat needle was used to punch the micro-hole array on a PDMS layer (3 mm thickness). Due to the elasticity and deformation of PDMS, the holes are a waist type with wide ends and a narrow middle (Figure 1F,G). The formation of droplets is shown in Figure 1. The arrangement of the droplet array could be easily controlled by the micro-hole’s position, which could be designed before the fabrication.

### 2.3. Tumor Spheroid Formation

The SimpleDrop chips were placed in the Petri dishes with the support of brackets. To maintain the necessary cell culture humidity, water was added to the bottom of the dishes. After rinsing the HS-SY-II monolayer cells with PBS and detaching them with 0.05% trypsin-EDTA (Invitrogen, Carlsbad, CA, USA), the HS-SY-II cells were resuspended in the culture medium to disperse for counting. Subsequently, the cell suspension was prepared at a concentration of approximately 5 × 10^4^ cells/mL in the culture medium. A 10 μL suspension (about 500 cells) was introduced into a micro-hole using a pipette, and a droplet formed under the chip. The cells gathered spontaneously into spheroids during cellular growth in a 37 °C incubator. The spheroids’ formation and morphology changes were observed and pictured daily using an optical microscope during a 9 day spheroid culture.

### 2.4. Evaluation of the Drug Effect

In this research, cisplatin (Sigma-Aldrich, St. Louis, MO, USA), paclitaxel (MCE) and methotrexate (MCE) were chosen as the model anticancer drugs. The drugs were dissolved in DMSO and stored in 1000× aliquots to avoid repeated freeze–thaw cycles. Subsequently, the stock solution was diluted with the culture medium to reach 10× the desired range of concentrations prior to use. A 1 μL quantity of corresponding drug solutions was added into each hole. Correspondingly, the same volume of the solvent was applied to the control group. The final concentrations of cisplatin were 10 μM, 20 μM and 40 μM, respectively. The effect of drugs with three concentrations on HS-SY-II spheroids was checked at 24 h, 48 h and 72 h. The final concentrations of paclitaxel and methotrexate were 10 nM, 20 nM and 40 nM, respectively. The concentrations of drugs were set according to the preliminary experimental results.

### 2.5. Cell Viability Test

To assess cell viability, Calcein AM (green for live cells, Solarbio) and propidium iodide (red for dead cells, Solarbio) staining was performed when the spheroids were exposed to the anticancer drugs for the indicated time. After treatment, the spheroids were transferred into a fresh culture plate (48 well). After being washed carefully with 1× assay buffer three times, the spheroids were stained with 2 µM Calcein AM solution and 4.5 µM of propidium iodide solution, incubated at 37 °C for 45 min and then the spheroids were imaged with a Leica DMi8 inverted fluorescence microscope (Leica Microsystems, Wetzlar, Germany).

### 2.6. Apoptosis Assay

The cell apoptosis was determined using a FITC Annexin V/PI apoptosis kit. After treatment with drugs, the spheroids were washed in 1× binding buffer and double-stained with Annexin V and PI for 30 min. Subsequently, a Leica DMi8 inverted fluorescence microscope (Leica Microsystems) was used to obtain fluorescence images.

### 2.7. Image Processing and Analyses

ImageJ software was used to merge the green and red fluorescence images and count the number of alive and dead cells. The data were analyzed using Graph PadPrism software.

## 3. Results

### 3.1. 3D Spheroid Formation

**Figure 2:**
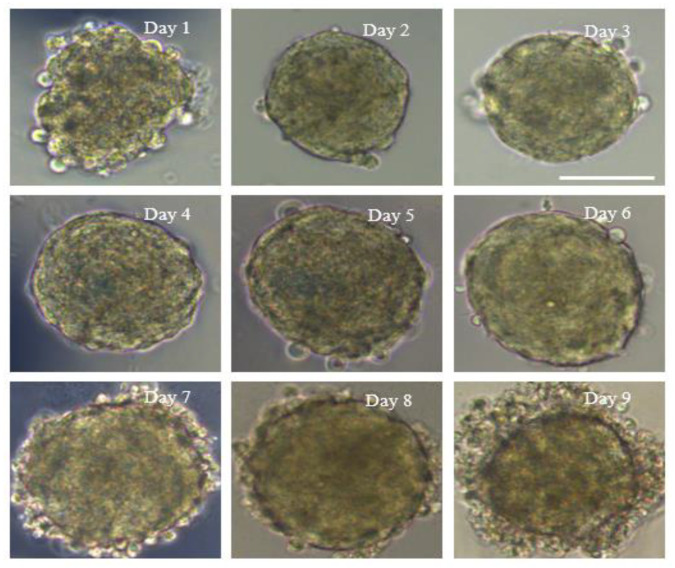
Morphology of multicellular 3D spheroid formation. The numbers on the pictures represent the culture day of the 3D spheroid. On day 1 after cell seeding, 3D spheroid formation occurred. On day 2, the diameter decreased slightly and the spheroid became tight and smooth. On days 3–6, the size of the spheroid gradually became larger. On days 7 and 8, the size displayed no significant change, but cells were observed detached from the edge of the spheroids. On day 9, a large number of cells were detached and the diameter of the spheroids decreased obviously. Magnification = 20×, Bar = 75 µm.

At the beginning, due to gravity, cells in droplets spontaneously formed aggregates at the bottom of the droplets and adhered to each other, and within 24 h spheroids appeared. After 48 h, the spheroids had smooth surfaces because the cells had aggregated firmly to form tight junctions. Subsequently, the size of the spheroids slightly reduced on day 2 compared to day 1, probably due to the tight inter-connection among the cells. Following this, they became larger over time during day 2 and day 6. On days 7 and 8, the size had no significant variation, but dead cells were observed on the surface of the spheroids. A large number of cells were detached from the spheroids on day 9 and the size of the aggregates was slightly reduced (Figure 2). In short, spheroid formation on the PDMS chips was composed of four stages: (I) within 24 h of incubation, cells aggregated into loose and irregular spheroids; (II) after seeding for 24 h, unaggregated cells were removed; (III) during days 3–6, spheroids formed and grew; (IV) on days 7–9, dead cells detached from the spheroids, which displayed a slight size reduction. Four independent experiments provided similar results. 

### 3.2. Effect of Anticancer Drugs on HS-SY-II 3D Spheroids

**Figure 3:**
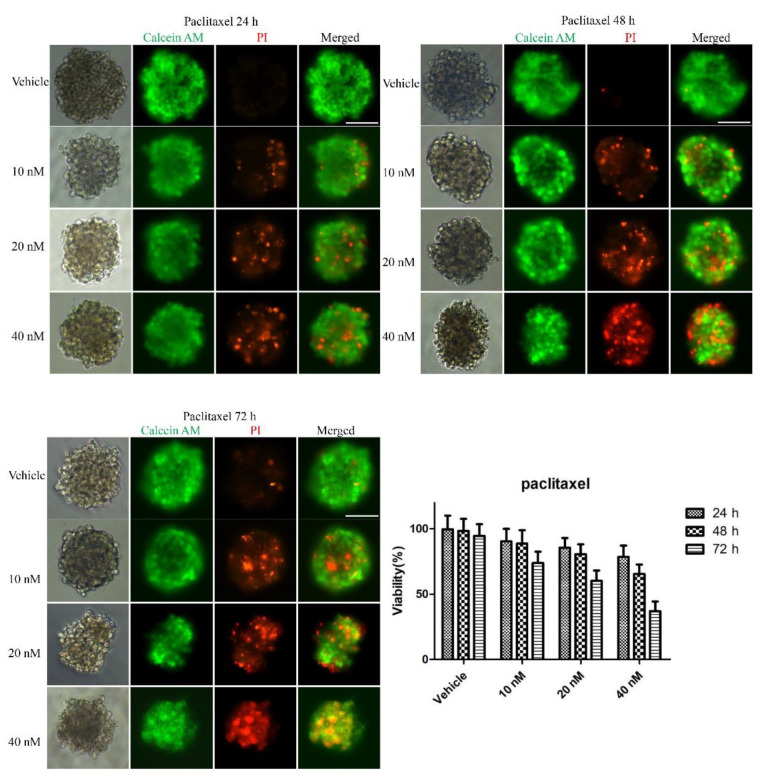
Drug assay (paclitaxel) on tumor spheroids: live/dead cell assay images stained with Calcein AM (green) for viable cells and PI (red) for dead cells. Cell viability decreased over time and concentration. The histogram shows the cell survival rate of the indicated concentrations after 24 h, 48 h and 72 h of the paclitaxel administration (the data are presented as mean ± SD). Magnification = 20×, Bar = 75 µm.

**Figure 4:**
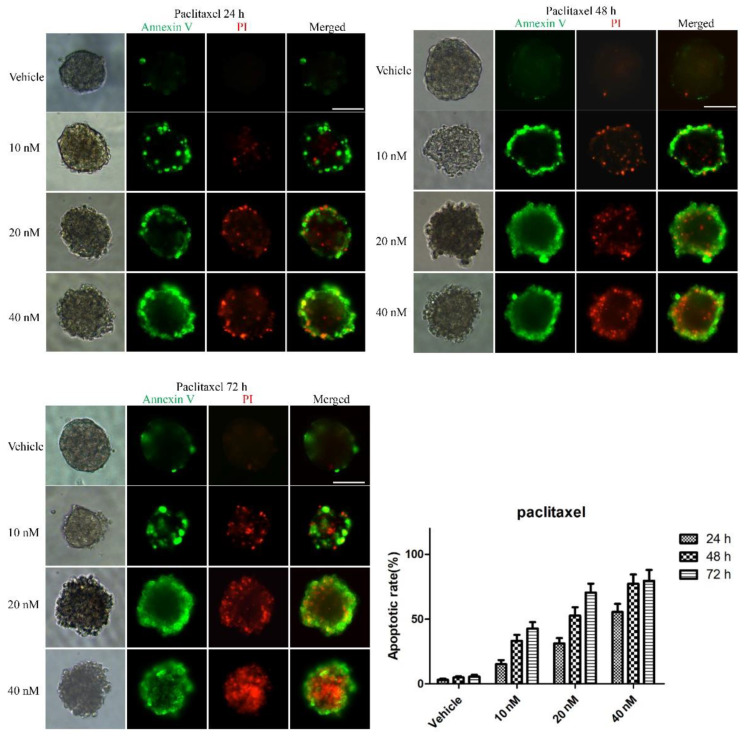
Immunofluorescence analysis of apoptotic marker (Annexin V, green) and necrotic marker (PI, red) after drug (paclitaxel) treatment in the 3D spheroid models. The fraction of the apoptotic and necrotic cells appeared to be correlated with the concentration and time. The histogram shows the apoptosis rate at the indicated concentrations after 24 h, 48 h and 72 h of the paclitaxel administration (the data are presented as mean ± SD). Magnification = 20×, Bar = 75 µm.

**Figure 5:**
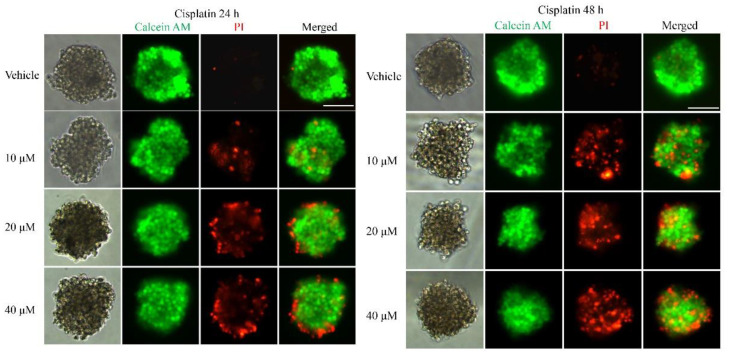

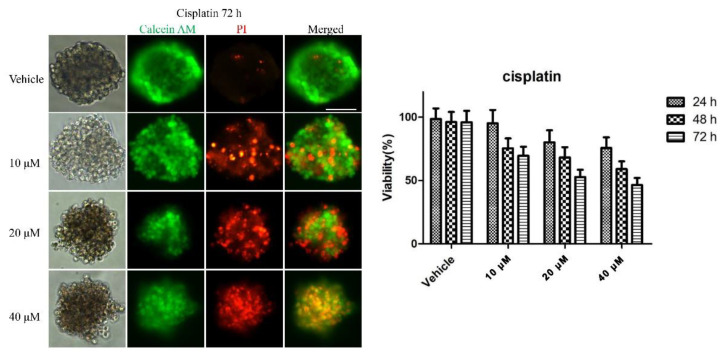
Drug assay (cisplatin) on tumor spheroids: live/dead cell assay images stained with Calcein AM (green) for viable cells or PI (red) for dead cells. Cell viability decreased over time and concentration. The histogram shows the cell survival rate of the indicated concentrations after 24 h, 48 h and 72 h of the cisplatin administration (the data are presented as mean ± SD). Magnification = 20×, Bar = 75 µm.

**Figure 6:**
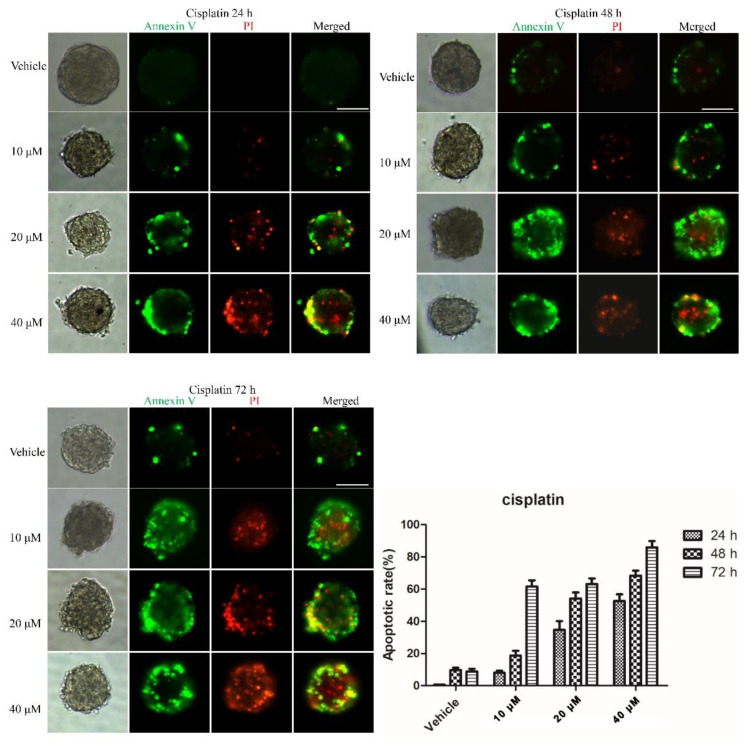
Immunofluorescence analysis of apoptotic marker (Annexin V, green) and necrotic marker (PI, red) after drug (cisplatin) treatment in the 3D spheroid models. The fraction of the apoptotic and necrotic cells appeared to be correlated with the concentration and time. The histogram shows the apoptosis rate at the indicated concentrations after 24 h, 48 h and 72 h of the cisplatin administration (the data are presented as mean ± SD). Magnification = 20×, Bar = 75 µm.

**Figure 7:**
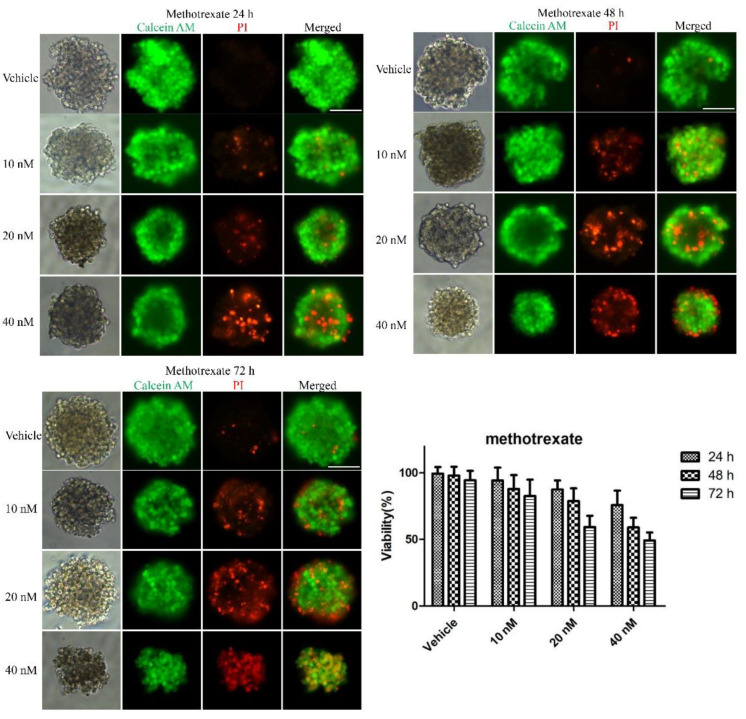
Drug assay (methotrexate) on tumor spheroids: live/dead cell assay images stained with Calcein AM (green) for viable cells or PI (red) for dead cells. Cell viability decreased over time and concentration. The histogram shows the cell survival rate of the indicated concentrations after 24 h, 48 h and 72 h of the methotrexate administration (the data are presented as mean ± SD). Magnification = 20×, Bar = 75 µm.

**Figure 8:**
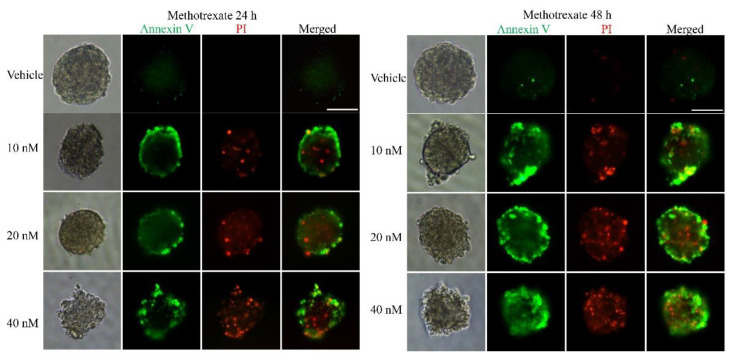

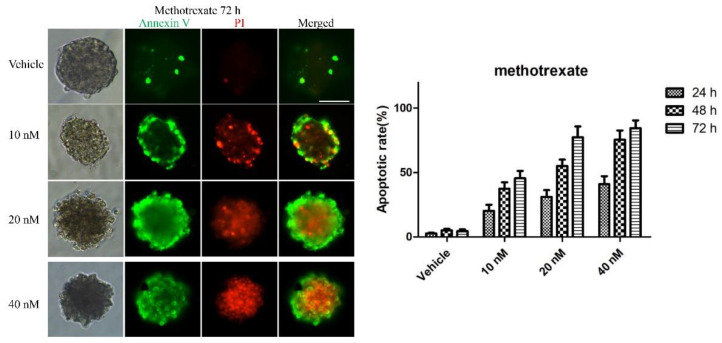
Immunofluorescence analysis of apoptotic marker (Annexin V, green) and necrotic marker (PI, red) after drug (methotrexate) treatment in the 3D spheroid models. The fraction of the apoptotic and necrotic cells appeared to be correlated with the concentration and time. The histogram shows the apoptosis rate at the indicated concentrations after 24 h, 48 h and 72 h of the methotrexate administration (the data are presented as mean ± SD). Magnification = 20×, Bar = 75 µm.

**Figure 9:**
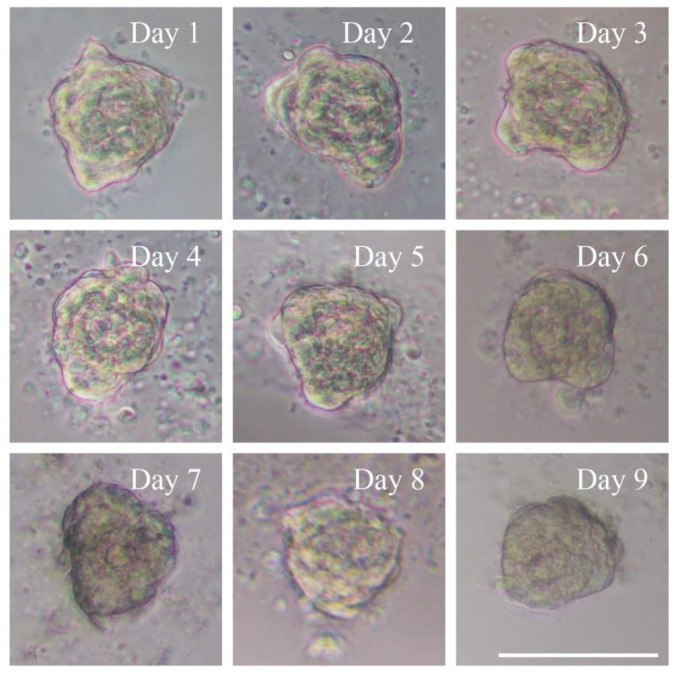
Morphology of multicellular 3D spheroids formation of UC-MSCs from day 1 to day 9. Bar = 100 µm.

To examine whether the 3D multicellular tumor spheroids formed in our system could provide a platform for the in vitro screening of drugs, drug testing was performed 48 h after seeding. Paclitaxel, as a natural anti-tumor drug, functions by stabilizing tubulin polymerization and causing both mitotic arrest and apoptotic cell death, and it has already been used for chemotherapy. In this work, three final concentrations of paclitaxel were applied: 10, 20 and 40 nM. After 24, 48, and 72 h, the tumor spheroids exhibited different results in response to different concentrations of paclitaxel. Figure 3 shows live/dead cell staining images after paclitaxel treatment. The results revealed the obvious death of tumor cells at the edge of the spheroids after 24 h of treatment. We also observed that the tumor cells demonstrated a dose-dependent response to paclitaxel (Figure 3). Specifically, the viability of the cells in higher concentrations was significantly less than that in lower concentrations of the drug. Similarly, a clear time-dependent trend indicated that the survival rate was inversely proportional to the treatment time. The histogram in Figure 3 displays the rates of the live cells cultured in paclitaxel of different concentrations for 24 h, 48 h and 72 h. The exact value is shown in Table 1 as mean ± SD. To further evaluate the dose-dependent cytotoxic effects of paclitaxel, the apoptosis of the cells was determined with FITC Annexin V/PI staining at the endpoint of each experiment. As shown in Figure 4, the cellular apoptotic and necrotic fraction in the higher drug concentration group showed a significant increase compared to the lower concentration, proving a paclitaxel-triggered apoptosis. At a concentration of 40 nM, paclitaxel showed the best penetration capacity into the spheroid and the largest number of apoptotic and necrotic cells. The histogram in Figure 6 displays the rates of the apoptotic and necrotic cells cultured in paclitaxel of different concentrations for 24 h, 48 h and 72 h. The exact value can be found in Table 2 as mean ± SD. The same pattern was found in the group of cisplatin/methotrexate-treated spheroids, indicating that the viability decreased over time and concentration, consistent with the well-established cytotoxic effect described by paclitaxel (as shown in Figure 5, Figure 6, Figure 7 and Figure 8). The corresponding viability rates and apoptotic and necrotic rates are presented in Table 1 and Table 2. To prove the validation of this SimpleDrop chip, UC-MSCs cellular spheroids were formed as shown in Figure 9.

## 4. Discussion

The SimpleDrop chip overcomes the shortcomings of the hanging drop method, making the medium replacement more convenient. Drug administration or factor addition into the culture medium is easy to operate. Moreover, unlike the spinner flask method, the drop method, having no shear force, avoids shear-force-induced damage to the 3D cell spheres. Furthermore, the punch-made micro-holes method avoids the need for any specialized chemical techniques and photosensitive materials as well as any complex processes for the preparation of micropatterned structures. Based on the merits of SimpleDrop chip, drug assays (paclitaxel, cisplatin and methotrexate) of 3D tumor spheres were easy to operate, and the results were easy to observe by fluorescence microscope. In addition, we verified the feasibility of robotic pipetting through existing data and related information. For example, Mehesz et al. used an EpMotion 5070 automated pipetting machine to seed cells into a 96-well plate. The results showed that the tissue spheroids made by this method are more uniform than those made by the conventional hanging drop method [40]. Gómez-Sjöberg et al. established an automatic cell culture and screening system based on a microfluidic chip that created arbitrary culture media formulations in 96 independent culture chambers and maintained cell viability for several weeks [41]. In the future, our SimpleDrop chip may extend the automation function to make this chip higher-throughput.

## 5. Conclusions

We propose an easy-to-operate and useful micro-hole culture chip, named the SimpleDrop chip, for the formation of 3D cell spheres, which has advantages over traditional methods in terms of its ease of operation, lack of shear force and environmental friendliness. On this chip, we clearly observed the formation of 3D spheroids. During 9 days’ culture, the morphology of the multicellular 3D spheroids presented several stages: they underwent aggregation, became tight and smooth, then became larger, stopped changing in size and finally some cells detached from the spheres. Subsequently, we processed the drug assay on tumor spheroids. Three drugs (paclitaxel, cisplatin and methotrexate) presented a similar conclusion: cell viability decreased over time and concentration. Moreover, the drug-induced apoptosis was tested. Immunofluorescence analysis of apoptotic and necrotic markers on the 3D spheroids showed similar trends with the live/dead cell assay, the fraction of the apoptotic and necrotic cells being correlated with the concentration and time. All these results prove that our SimpleDrop method is a useful and easy method for the formation of 3D cellular spheroids, demonstrating its potential for cell–cell interaction research, tissue engineering and anticancer drug screening.

## Figures and Tables

**Table 1:** Summary of the cell survival rate at the indicated concentrations 24 h, 48 h and 72 h after the drug’s administration.

Drugs	Concentration	Time		
		24 h	48 h	72 h
Paclitaxel	Vehicle	99.6 ± 10.54%	98.3 ± 9.32%	94.7 ± 8.90%
	10 nM	86.3 ± 9.67%	74.9 ± 10.29%	61.5 ± 8.66%
	20 nM	77.6 ± 7.39%	61.8 ± 7.49%	44.8 ± 7.85%
	40 nM	64.8 ± 7.04%	55.2 ± 7.04%	20.2 ± 7.37%
Cisplatin	Vehicle	98.6 ± 8.35%	95.0 ± 7.98%	96.0 ± 9.01%
	10 μM	88.4 ± 9.53%	68.6 ± 7.89%	58.5 ± 7.03%
	20 μM	79.6 ± 9.47%	68.2 ± 8.12%	37.7 ± 5.79%
	40 μM	56.3 ± 8.31%	45.0 ± 6.19%	19.3 ± 5.63%
Methotrexate	Vehicle	99.4 ± 0.07%	98.1 ± 6.55%	94.6 ± 6.90%
	10 nM	93.5 ± 9.75%	64.1 ± 5.58%	56.8 ± 3.25%
	20 nM	79.2 ± 6.89%	55.0 ± 6.60%	41.0 ± 2.45%
	40 nM	69.2 ± 7.81%	50.0 ± 6.23%	33.0 ± 2.07%

**Table 2:** Summary of the cell apoptosis rate at the indicated concentrations 24 h, 48 h and 72 h after the drug’s administration.

Drugs	Concentration	Time		
		24 h	48 h	72 h
Paclitaxel	Vehicle	3.1 ± 0.86%	4.8 ± 0.91%	5.5 ± 1.45%
	10 nM	15.1 ± 3.26%	33.2 ± 4.65%	42.7 ± 5.00%
	20 nM	31.1 ± 4.23%	52.8 ± 6.44%	70.7 ± 6.85%
	40 nM	55.6 ± 6.27%	77.4 ± 7.04%	79.5 ± 8.45%
Cisplatin	Vehicle	0.6 ± 0.07%	9.7 ± 2.54%	8.8 ± 3.03%
	10 μM	8.0 ± 2.21%	18.7 ± 5.22%	61.7 ± 6.55%
	20 μM	34.7 ± 9.43%	54.2 ± 6.48%	63.2 ± 5.77%
	40 μM	52.6 ± 5.03%	68.3 ± 5.38%	85.9 ± 6.77%
Methotrexate	Vehicle	2.7 ± 0.54%	5.2 ± 1.02%	4.5 ± 1.33%
	10 nM	20.2 ± 4.75%	37.4 ± 5.01%	45.6 ± 5.67%
	20 nM	31.0 ± 5.34%	55.0 ± 5.09%	77.3 ± 8.45%
	40 nM	40.9 ± 6.24%	75.4 ± 7.23%	84.4 ± 6.07%

## Data Availability

Data sharing not applicable. No new data were created or analyzed in this study. Data sharing is not applicable to this article.
